# Content of Care in 15,000 Sick Child Consultations in Nine Lower‐Income Countries

**DOI:** 10.1111/1475-6773.12842

**Published:** 2018-03-07

**Authors:** Margaret E. Kruk, Anna D. Gage, Godfrey M. Mbaruku, Hannah H. Leslie

**Affiliations:** ^1^ Department of Global Health and Population Harvard T.H. Chan School of Public Health Boston MA; ^2^ Ifakara Health Institute Dar es Salaam Tanzania

**Keywords:** Health care quality, child mortality

## Abstract

**Objective:**

Describe content of clinical care for sick children in low‐resource settings.

**Data Sources:**

Nationally representative health facility surveys in Haiti, Kenya, Malawi, Namibia, Nepal, Rwanda, Senegal, Tanzania, and Uganda from 2007 to 2015.

**Study Design:**

Clinical visits by sick children under 5 years were observed and caregivers interviewed. We describe duration and content of the care in the visit and estimate associations between increased content and caregiver knowledge and satisfaction.

**Principal Findings:**

The median duration of 15,444 observations was 8 minutes; providers performed 8.4 of a maximum 24 clinical actions per visit. Content of care was minimally greater for severely ill children. Each additional clinical action was associated with 2 percent higher caregiver knowledge.

**Conclusions:**

Consultations for children in nine lower‐income countries are brief and limited. A greater number of clinical actions was associated with caregiver knowledge and satisfaction.

Despite recent global declines, child mortality remains high in low‐ and middle‐income countries. Over 1.7 million children died of pneumonia, diarrhea, and malaria in 2015 (Liu et al. 2015, 2016); the majority of these deaths could be averted through prevention and appropriate clinical care. International donors and governments invest considerable development assistance funding in programs to improve health, including health system strengthening: $37.6 billion in 2016 across all donors and nearly $6 billion in bilateral funding from the United States alone (Dieleman et al. 2017). Investments for newborn and child survival increased by over 10 percent per year between 2000 and 2009.

Despite the increased funding, challenges in providing care abound. Shortages of physicians in low‐income countries have led to task shifting of care of sick children to paraprofessionals, such as clinical officers and nurses. To ensure minimum quality standards across a range of health workers, the World Health Organization developed the Integrated Management of Childhood Illness, a protocol for identifying and managing common childhood illness intended for use by a range of health care workers with proven effectiveness in improving child health outcomes (Bryce et al. 2005). However, despite the existence of protocols, quality of clinical care for sick children remains weak in many low‐income countries (Gera et al. 2016). To support uptake of protocols, global funders and national governments invested heavily in in‐service training courses, but this too has had limited effect on quality of care (Leslie et al. 2016).

While provider knowledge of treatment guidelines is not optimal, practice is often observed to be even worse, a so‐called know‐do gap (Mohanan et al. 2015). One mechanism for the gap may be low provider effort and time spent in the clinical consultations (Lange, Mwisongo, and Maestad 2014). Low provider effort combined with resource constraints may result in cursory interactions with low content of clinical care (Mohanan et al. 2015). Inadequate history and physical examination may lead to incorrect diagnoses and inappropriate treatment. Failing to inform parents of the diagnosis and signs of complications may harm their ability to care for their children and deter follow‐up. Nonetheless, few studies of content of care exist (Das and Gertler 2007). As the main causes of death in low‐income countries shift from readily prevented and treated illness (e.g., diarrhea) to more complex conditions (e.g., neonatal sepsis, pneumonia), poor clinical quality will limit reductions in child mortality (Kruk, Larson, and Twum‐Danso 2016).

In this article, we investigate visit duration and content of care provided during sick child consultations, variation across key stratifiers, and effects on caregiver knowledge and satisfaction.

## Methods

While routine health data such as vital statistics registries and health information systems are sparse in low‐income, high‐mortality countries, detailed health system surveys have taken place in multiple lower‐income countries. Service provision assessments are comprehensive health facility assessments that include direct observation of outpatient consultations for sick children under age 5. Countries conduct a census or select a representative sample of health facilities. A facility infrastructure assessment is conducted and is accompanied by clinical observations of care and exit interviews with patients. Within a facility, children presenting with illness are enumerated and sampled using systematic random sampling with the goal of sampling up to five observations per provider and a maximum of 15 total. Direct observations are conducted by trained assessors (typically nurses) on the survey team; caretakers for all observed children are approached for an exit interview.

We pooled data from Service Provision Assessments in nine countries: Haiti (census, 2013), Kenya (2010), Malawi (census, 2013), Namibia (census, 2009), Nepal (2015), Rwanda (near census, 2007), Senegal (2013–2015), Tanzania (2015), and Uganda (2007) (The DHS Program 2015). We excluded each provider's first observed consultation to minimize the Hawthorne effect (Leonard and Masatu 2006) and excluded children admitted to the facility as these may have received additional clinical assessments after the observed interaction.

Donabedian proposed that quality of care be assessed through structure, processes, and outcomes (Donabedian 1988). Much of the data on quality in low‐ and middle‐income countries have focused on structure (e.g., medicines, equipment, staff, provider knowledge; Nickerson et al. 2015). Here, we examine a process measure of quality: the content of clinical care. We calculated visit duration in minutes (excluding visits with conflicting time information and the longest 1 percent of visits) and a care content index that measures performance of recommended clinical actions in three domains (max 24). History contained nine items, for example “asked if child had a cough,” the physical included 10 items, for example “took temperature,” and counseling included five items, for example “provider discussed follow‐up visit.” We did not assess whether these actions were appropriate to the presenting case, only their total number.

We selected key visit attributes at the facility, provider, and child levels, including private versus public facility, service readiness of the facility (an index covering five domains—basic amenities, basic equipment, infection prevention measures, diagnostics, and medications) as defined by the World Health Organization (WHO; World Health Organization 2013), cadre of health worker (e.g., physician vs. registered nurses), patient volume on day of assessment, whether the child was severely ill, defined as caretaker report of convulsions or both inability to eat or drink and vomiting everything following WHO guidelines for Integrated Management of Childhood Illness (World Health Organization 2014), and diagnosis provider assigned. Total patient volume was calculated based on a count of children waiting for care on the day of the assessment; load per provider divided patient volume by the number of providers directly observed. We classified provider diagnoses as none, gastrointestinal, respiratory, fever/malaria, multiple of these three, or other, which included ear and skin conditions.

We also defined two important outcomes of every visit. Caretaker knowledge regarding child's illness and treatment was defined as the percent of responses to five items: knowledge of at least one danger sign requiring a return to care, knowledge of nonemergency reasons for return to care (e.g., medication refill or follow‐up visit), knowledge of appropriate feeding and of appropriate fluids to provide during the illness, and confidence in providing the appropriate dose and duration of medication (if any). Satisfaction was defined as responding “very satisfied” regarding services provided at the facility that day. Patient satisfaction is an important objective of the health system in and of itself (World Health Organization 2000; Roberts et al. 2003) as well as a predictor of utilization of health services, choice of provider, adherence to prescribed regimens, and better clinical outcomes. (Fan et al. 2005; Glickman et al. 2010; Boulding et al. 2011; Jerant et al. 2014).

We report descriptive statistics for each covariate and outcome of interest, including median and interquartile range (IQR) for nonnormally distributed continuous variables. We compare content of care across key predictors of private versus public facility, provider cadre, sicker children, top quartile of patient load within each country, and diagnosis given. We tested differences using linear regression accounting for clustering within facilities and report within‐country differences significant at *p* ≤ .05.

To evaluate the association between care content and outcomes, we first visually assessed the form of each exposure; we categorized duration into quartiles (<4 minutes, 5–8 minutes, 9–12 minutes, over 12 minutes) to better capture the nonlinear relationships between duration and each outcome. We then regressed each outcome on duration and care content in turn and together, controlling for factors that could influence provider behavior and parents’ expectations of care: child's illness severity, caretaker's education level, and service readiness of the facility. We included an indicator variable for each survey country. We used generalized estimating equations clustered at the facility level and report robust standard errors to account for heteroskedasticity (nonconstant variance) of residuals. Observations from Rwanda and Uganda were excluded from the satisfaction analysis as this question was not included on the survey in these countries.

All analyses were performed in *STATA* 14.1 (StataCorp, College Station, Texas, USA); descriptive analyses include sampling weights. The Harvard University Human Research Protection Program approved this analysis of secondary, deidentified data as exempt from human subjects review.

## Results

Of 23,005 sick child consultations observed across the study countries, 15,444 visits in 4,717 facilities met inclusion criteria. Under‐five mortality in study countries ranged from 38 per 1,000 in Nepal to over 100 per 1,000 in Uganda at the time of each survey, while human resources for health are sparse (Table 1). Five of the study countries—Malawi, Nepal, Rwanda, Senegal, and Tanzania—have fewer than 10 physicians per 100,000 people. Children were seen in facilities with consistent gaps in essential service readiness (from 45 percent of expected readiness in Uganda to 73 percent in Namibia) and relatively low patient load per provider. The median number of children per provider was 3 in Nepal and 13 in Malawi, with an overall median of 5 and IQR from 3 to 9. The majority of visits (76 percent) occurred at public facilities; a minority of children (14 percent) were seen by physicians. Eleven percent of children were classified as severely ill. Caretakers were generally satisfied with care (76 percent reported being very satisfied on average), although knowledge of appropriate management for their child was low, with an average of 37 percent.

**Table 1:** Health System Characteristics and Content of Care during Sick Child Visits in Nine Low‐ and Middle‐Income Countries (*N* = 15,444)

	Haiti 2013	Kenya 2010	Malawi 2013	Namibia 2009	Nepal 2014	Rwanda 2007	Senegal 2013–2015	Tanzania 2015	Uganda 2007	Nine Country Average
*Country characteristics* a										
Under‐5 mortality per 1,000 live births	73.1	62.2	68.7	58.6	38.0	89.5	55.5	58.8	100.5	NA
Physicians per 100,000	–	17.7	1.9	37.4	–	5.5	5.9	3.0	11.7	NA
Gross domestic product per capita (2010 USD)	718.4	967.3	471.8	4,978.7	675.7	482.1	1,007.4	836.0	538.0	NA
*Facility characteristics*										
Facilities	509	442	674	275	481	371	819	885	261	4,717
Service readiness^b^ (mean ± SD)	60% ± 16%	58% ± 16%	58% ± 16%	73% ± 8%	49% ± 14%	64% ± 12%	63% ± 10%	49% ± 17%	45% ± 16%	58% ± 16%
Patients per provider (Median [IQR])^c^	5 (3–9)	5 (3–10.5)	13 (6–25)	5 (3–7)	3 (2–5)	6 (4–10)	5 (3–7)	5 (3–6)	6 (3–10)	5 (3–9)
*Sick child visit characteristics*										
Sick child visits observed	1,617	1,291	2,437	1,084	1,322	1,210	2,502	3,333	648	15,444
% at private facility	60%	24%	22%	12%	16%	35%	17%	16%	9%	24%
% at hospital	35%	24%	35%	6%	38%	5%	6%	12%	8%	19%
Age										
<2 months	8%	1%	3%	3%	5%	4%	6%	4%	3%	4%
2–11 months	31%	28%	34%	28%	26%	31%	33%	35%	34%	31%
12–59 months	61%	71%	62%	69%	69%	65%	61%	61%	63%	65%
% with provider type^d^										
MDs	69%	2%	1%	4%	35%	6%	10%	3%	1%	14%
Associate clinicians^e^	NA	34%	86%	NA	55%	NA	31%	62%	39%	34%
RNs	14%	17%	1%	40%	10%	2%	24%	8%	4%	13%
Other nurses^j^	17%	46%	13%	56%	0%	92%	35%	27%	57%	38%
% where child is severely ill^f^	16%	21%	8%	9%	8%	12%	5%	9%	14%	11%
*Diagnosis*										
None	7%	1%	1%	6%	9%	3%	3%	3%	1%	4%
Diarrhea, digestive disorder, dehydration	14%	4%	10%	9%	18%	7%	18%	11%	1%	10%
Fever, malaria	10%	16%	18%	4%	17%	14%	3%	25%	21%	14%
Respiratory illness	17%	26%	40%	41%	23%	11%	29%	28%	14%	26%
More than one of the above	14%	46%	13%	23%	3%	59%	6%	20%	58%	27%
Other (e.g., ear infection)	38%	8%	18%	16%	29%	6%	41%	14%	5%	19%
% where caretaker is very satisfied with care received^i^	92%	79%	82%	75%	42%	NA	91%	74%	NA	76%
Caretaker knowledge on exit^g^ (mean ± SD)	41% ± 25%	42% ± 24%	34% ± 21%	33% ± 30%	40% ± 25%	21% ± 28%	38% ± 25%	38% ± 21%	41% ± 36%	37% ± 27%
*Visit characteristics*										
Duration of visit in minutes (*N* = 12,825) Median (IQR)	10 (6–13)	8 (5–13)	6 (4–14)	10 (7–14)	6 (4–9)	6 (5–10)	9 (7–13)	12 (8–19)	6 (4–10)	8 (5–12)
Content of care (max 24 items) *N* (%)^h^	7.7 (32%)	10.4 (43%)	6.8 (28%)	11.6 (49%)	6.3 (26%)	7.8 (33%)	7.7 (32%)	7.2 (30%)	10.6 (45%)	8.4 (35%)
History items (max 9)	3.0 (33%)	4.4 (49%)	2.9 (32%)	4.5 (51%)	2.6 (29%)	3.0 (34%)	3.0 (34%)	3.8 (43%)	5.0 (56%)	3.6 (40%)
Examination items (max 10)	3.6 (36%)	3.8 (38%)	2.3 (23%)	4.5 (45%)	2.3 (23%)	3.5 (36%)	3.4 (34%)	2.1 (21%)	3.7 (37%)	3.3 (33%)
Counseling items (max 5)	1.1 (23%)	2.2 (44%)	1.6 (31%)	2.6 (53%)	1.3 (26%)	1.3 (26%)	1.3 (26%)	1.2 (25%)	1.9 (39%)	1.6 (33%)

a From World Development Indicators using data from year of health facility survey or closest available year.

b Facility service readiness index calculated as average performance in five domains (basic amenities, basic equipment, infection control measures, diagnostics, medication) as defined by the World Health Organization.

c Calculated as total patients observed on day of assessment over total staff directly observed providing sick childcare.

d Not all provider type options are available in every country.

e Associate clinicians include advanced practice clinicians (e.g., assistant medical officers) and paramedical (e.g., clinical officers).

f Severe illness is defined as child with recent convulsions or child refusing food and vomiting everything.

g Caretaker knowledge based on response to five items on exit interview: child's diagnosis, danger signs requiring return, plan for follow‐up visit, and (for those with medication prescribed) how to give medication and appropriate dose.

h Content of care is the sum of history, examination, and counseling items. Percent is average number of items completely divided by the maximum.

i Nepal was the only country to offer five response options for satisfaction: very satisfied, fairly satisfied, neutral, fairly dissatisfied, very dissatisfied. All other surveys offered three options: very satisfied, more or less satisfied, not satisfied.

j Includes enrolled nurses and nursing aides and infrequently pharmacy and other technicians, community health agents and other non‐clinical staff.

Median visit length ranged from six minutes (Malawi, Nepal, Rwanda, Uganda) to 12 minutes in Tanzania; 82 percent of visits were shorter than 15 minutes. The mean content of care index was one‐third of total possible items, with poor performance across history, examination, and counseling domains. Performance by item for the full sample is shown in Figure S1 in Appendix SA2. Providers in Kenya, Namibia, and Uganda performed the most thorough assessments, yet the average provider in these countries still completed fewer than half of the index items. Visit duration was positively correlated with number of items completed, though not strongly so (Pearson's correlation coefficient= 0.19). Table S1 in Appendix SA2 provides average duration and care content by child and facility characteristics.

**Figure 1:**
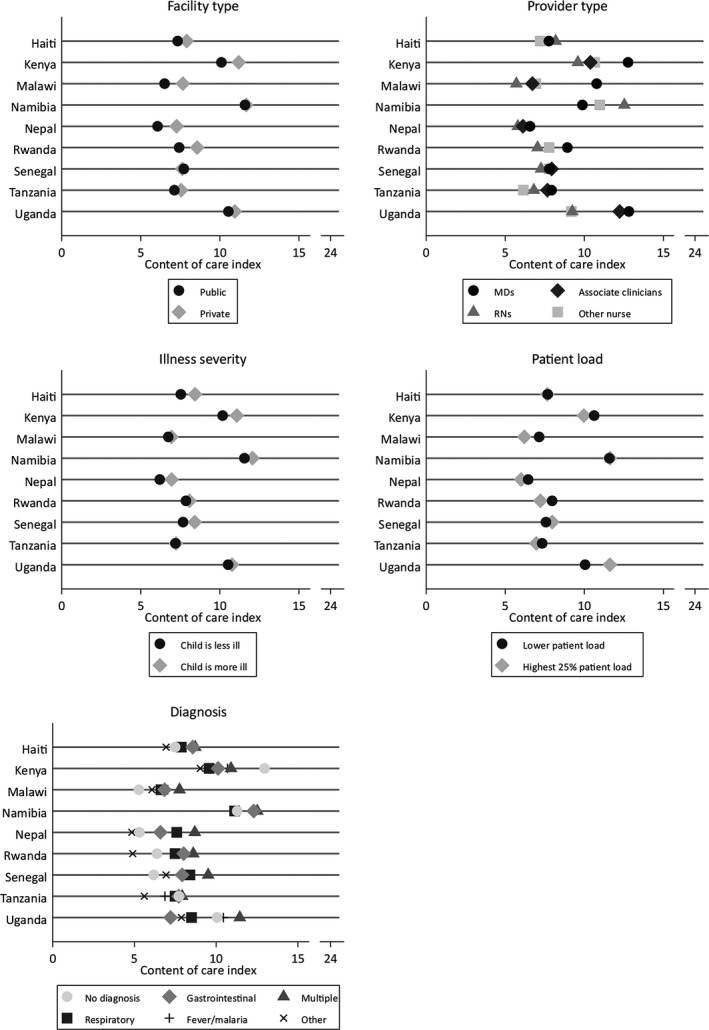
Content of Care Index Stratified by Facility Type, Provider Type, Severity of Illness, Patient Load, and Diagnosis Assigned in Nine Countries

*Note*. Not all provider type options are available in every country. Associate clinicians include advanced practice clinicians (e.g., assistant medical officers) and paramedical (e.g., clinical officers). Severe illness is defined as child with recent convulsions or child refusing food and vomiting everything. Content of care index is sum of history, examination, and counseling items (maximum 24).

Assessment of content of care by key stratifiers shown in Figure 1 reveals varying patterns by country. Visits included more content in private facilities in four countries; physicians outperformed the average across other cadres in three of nine countries. In three countries, providers performed approximately one additional index item for severely children in comparison to nonseverely ill children. Higher patient load (minimum of six patients in Nepal, 26 in Malawi—see upper limit of IQR in Table 1 for per‐country threshold) was significantly associated with less content of care only in Malawi, the country with highest observed volume. In all countries, visits that resulted in multiple diagnoses included more clinical actions than other visits. Visits that did not result in any diagnosis contained fewer clinical actions.

Regression models (Table 2) support an association between content of care and caretaker outcomes, particularly knowledge. Each additional clinical action was associated with 2 percent greater knowledge. Caretaker scores on the knowledge index were 10 percent higher after visits over 12 minutes than visits under 4 minutes long. These associations were smaller but still significant in the fully adjusted model three, indicating that duration and content of care have independent associations with caretaker knowledge. Visit duration was not significantly associated with caretaker satisfaction, but caretakers had higher odds of being very satisfied when content of care was more extensive (adjusted odds ratio 1.04 for each additional clinical action).

**Table 2:** Association of Care Content and Duration with Client Knowledge and Satisfaction

A: Client Knowledge *N* = 13,205	Model 1 Beta (95% CI)	Model 2 Beta (95% CI)	Model 3 Beta (95% CI)
Care content (*N* items)	**2.00 (1.87, 2.14)**		**1.87 (1.73, 2.01)**
*Duration*			
<4 minutes		1.00 (REF)	1.00 (REF)
5–8 minutes		**3.07 (1.85, 4.29)**	1.07 (−0.13, 2.28)
9–12 minutes		**6.15 (4.73, 7.57)**	**2.44 (1.03, 3.85)**
>12 minutes		**10.36 (8.95, 11.77)**	**5.27 (3.86, 6.69)**

B: Satisfaction *N* = 11,556	Model 1 AOR (95% CI)	Model 2 AOR (95% CI)	Model 3 AOR (95% CI)
Care content (*N* items)	**1.04 (1.02, 1.05)**		**1.04 (1.02, 1.05)**
*Duration*			
<4 minutes		1.00 (REF)	1.00 (REF)
5–8 minutes		0.96 (0.83, 1.11)	0.92 (0.79, 1.06)
9–12 minutes		1.06 (0.90, 1.25)	0.98 (0.83, 1.15)
>12 minutes		1.11 (0.95, 1.29)	0.99 (0.84, 1.17)

*Note*. All models adjusted for child's illness severity, caretaker's education level, and service readiness of the facility as well as a fixed effect for survey country. Caretaker satisfaction was not assessed in the Rwanda and Uganda surveys; these observations are excluded from the satisfaction analysis. Bold values indicate significance at alpha = 0.05.

## Discussion

The quality of clinical care provided in a sick child visit is a function of the provider's knowledge, his/her effort in applying that knowledge, and institutional incentives and constraints to high‐quality care, yet few studies have examined the content and duration of care in low‐income countries (Das and Gertler 2007). Using a unique cross‐national dataset of clinical observations, we found that sick child consultations were brief (median of 8 minutes) and limited (an average of eight history, physical examination, and counseling actions, vs. 24 recommended actions). Similarly, poor performance has been identified in a recent smaller study (Edward et al. 2016). This weak clinical assessment may in part be responsible for poor diagnostic accuracy and high rates of incorrect treatment identified in some of the study countries (Zurovac and Rowe 2006).

The slightly higher content of care in private clinics (typically mission facilities) in some countries may be due to higher remuneration, a more conducive clinical environment, or higher expectations from clinic managers. Private facilities performed better for antenatal and sick childcare after adjustment for staff and infrastructure in a study of seven sub‐Saharan African countries (Kruk et al. 2017). Haiti and Nepal were the only study countries where physicians provided a substantial amount of the care; however, physicians did not perform better than associate clinicians and nurses in these countries. Overall differences between physicians and other health care workers were marginal.

A concerning finding was that the number of clinical actions was at most only marginally higher in consultations involving very ill children. These children are at high risk for adverse outcomes and require systematic assessment to determine the correct clinical course. Content of care differed little by diagnosis. The limited clinical performance for acute respiratory infection we found may result in failure to detect pneumonia, which in turn contributes to high mortality rates for children from this disease (Adegbola 2012; Enarson et al. 2014).

How do these results compare to other settings? Nationally representative data from the United States suggest that in 2010, the average primary care physician visit for children was nearly 18 minutes long; a greater number of correct diagnoses was associated with longer visits (Shaw et al. 2014). Although there are many differences in the context of care between the United States and study countries, the nearly two‐fold difference in visit duration supports the conclusion of limited clinical value provided to children in these brief encounters with the health care system.

There are limited data on the content of clinical assessment in low‐income countries, but a study in Cambodia, Guatemala, Kenya, and Zambia found that consultations lasting more than 10 minutes were associated with more thorough assessment compared to shorter visits (Edward et al. 2016). A recent study in Zambia found that none of the children presenting to outpatient clinics in Zambia were assessed for all seven signs and symptoms of febrile illness and that fewer than half of those with pneumonia received the appropriate antibiotic (Lunze et al. 2017). Studies in Africa showed that quality was not improved through brief trainings or supervision, but a large, longitudinal study in Afghanistan found positive effects from a systematic investment in doctors and well‐trained health workers as well as facility structures and management (Zurovac et al. 2004; Osterholt et al. 2006; Edward et al. 2012).

The study had several limitations. First, the data did not permit us to gauge diagnostic or treatment accuracy or patient outcomes; it is possible to arrive at a correct diagnosis without a thorough examination. Second, it is difficult to separate knowledge from effort and other factors in understanding content of care. However, the assessment of a sick child is relatively formulaic it should be very familiar to health providers in the study countries, and there has been extensive recent training to improve practice in these settings (Gera et al. 2016). In addition, other research has found that providers frequently know more than they practice (Mohanan et al. 2015); it is unlikely that knowledge was the sole constraint to a more thorough clinical assessment. To ensure we were not underestimating content of care, we gave providers credit for all performed clinical actions, whether required for the case or not. Lastly, the findings are applicable only to the study countries.

We found low performance of basic clinical tasks in the care of sick children in nationally representative data from countries with high rates of child mortality and significant global investment in health‐related assistance. While we were not able to gauge accuracy of diagnosis or treatment in the consultations, it is unlikely that highly effective care could consistently be delivered with this level of clinical assessment, particularly to severely ill children and those with less common conditions. There is an urgent need for systematic research on the quality of care provided in health care facilities, especially as utilization of health care continues to rise. The reasons for poor quality of care need to be investigated, beginning with the level of clinical preparation and motivation among doctors and nurses. As the disease burden shifts to more complex conditions in lower‐income countries, mortality is unlikely to decline further without greater attention to issues such as care content.

## Supporting information

Appendix SA1: Author Matrix.

Appendix SA2: Supplementary Material.
Figure S1: Frequency of Performance of Items in Content of Care Index (*N* = 15,444 Observations).
Table S1: Content of Care and Duration of Visits by Child Characteristics.
