# Supplementary material for: Content of Care in 15,000 Sick Child Consultations in Nine Lower‐Income Countries
Source: Health Serv Res. 2018 Mar 7;53(4):2084–98. doi: 10.1111/1475-6773.12842 (PMC6052007; doi:10.1111/1475-6773.12842)
Supplement: Supplementary file 2 — Appendix SA2: Supplementary Material. Figure S1: Frequency of Performance of Items in Content of Care Index (N = 15,444 Observations). Table S1: Content of Care and Duration of Visits by Child Characteristics. [file HESR-53-2084-s002.docx]

Supplemental material

Figure S1: Frequency of performance of items in content of care index (N=15,444 observations)

Table S1: Content of care and duration of visits by child characteristics

|  | Content of care (N items) | | Duration (minutes) | |
| --- | --- | --- | --- | --- |
|  | N | Mean ± SD | N | Mean ± SD |
| Age |  |  |  |  |
| < 2 mo. | 689 | 8.0 ± 4.0 | 630 | 10.7 ± 8.5 |
| 2 mo. – 11 mo. | 4972 | 8.8 ± 4.1 | 4352 | 10.4 ± 9.0 |
| 12 mo. – 59 mo. | 9783 | 8.3 ± 4.2 | 8338 | 9.9 ± 8.1 |
| Gender |  |  |  |  |
| Male | 7953 | 8.5 ± 4.3 | 6859 | 10.2 ± 8.2 |
| Female | 7455 | 8.4 ± 4.1 | 6427 | 10.1 ± 8.6 |
| Facility type |  |  |  |  |
| Non-hospital | 11866 | 8.6 ± 4.3 | 10320 | 10.2 ± 8.2 |
| Hospital | 3578 | 7.8 ± 3.8 | 3000 | 9.6 ± 9.1 |
| Diagnosis |  |  |  |  |
| None | 526 | 7.5 ± 4.3 | 470 | 10.0 ± 8.2 |
| Gastroentestinal | 1809 | 8.2 ± 3.9 | 1551 | 10.1 ± 7.6 |
| Fever/malaria | 2181 | 8.1 ± 4.1 | 1689 | 11 ±10.3 |
| Respiratory | 4283 | 8.4 ± 3.8 | 3709 | 10.2 ± 8.2 |
| Multiple | 3303 | 10.0 ± 4.4 | 2844 | 10.0 ± 8.7 |
| Other | 3342 | 6.8 ± 4.1 | 3057 | 9.6 ± 7.2 |
